# Safety and efficacy of lumefantrine-artemether (Coartem^®^) for the treatment of uncomplicated *Plasmodium falciparum *malaria in Zambian adults

**DOI:** 10.1186/1475-2875-5-73

**Published:** 2006-08-21

**Authors:** Modest Mulenga, Jean-Pierre Van geertruyden, Lawrence Mwananyanda, Victor Chalwe, Filip Moerman, Roma Chilengi, Chantal Van Overmeir, Jean-Claude Dujardin, Umberto D'Alessandro

**Affiliations:** 1 Department Parasitology, Institute of Tropical Medicine, Nationalestraat 155, B-2000 Antwerp, Belgium; 2 Clinical Department, Institute of Tropical Medicine, Nationalestraat 155, B-2000 Antwerp, Belgium; 3 Department of Clinical Sciences, Tropical Disease Research Center, Ndola, Zambia

## Abstract

**Background:**

In Zambia, unacceptably high resistance to commonly used antimalarial drugs prompted the choice of artemether-lumefantrine (AL) as first line treatment for uncomplicated *Plasmodium falciparum *malaria. Although the safety and efficacy of AL have been extensively documented, no clinical trials had been carried out in Zambia.

**Methods:**

Nine hundred seventy one adult patients with uncomplicated malaria were randomized to either sulfadoxine-pyrimethamine (SP)(486) or AL (485) and followed up for 45 days. Outcome of treatment was defined according to the standard WHO classification. Recurrent parasitaemia were genotyped to distinguish between recrudescence and new infection.

**Results:**

Fever at day 3 was significantly lower (AL: 0.9%; 4/455; SP: 3,5%; 15/433; p = 0.007) and the mean haemoglobin at day 45 significantly higher (AL: 134 g/l; SP 130 g/l; p = 0.02) in the AL group. Almost all clinical symptoms cleared faster with AL. Early treatment failure was significantly higher in the SP (25/464) than in the AL (2/463) (OR: 13.1 95% CI: 3.08–55.50; P < 0.001). The rate of new infections was similar in both groups (18 with SP and 19 with AL). Late clinical failure (OR: 2.55; 95% CI: 1.34–4.84; P = 0.004) and late parasitological failure (OR:3.18; 95% CI: 1.25–8.09; P = 0.02) were significantly higher in the SP group. Total treatment failure was significantly higher in the SP group (96/393; 19.3%) as compared to the AL (22/403; 5.4%) group (OR: 4.15; 95% CI: 2.52–6.83; P < 0.001).

**Conclusion:**

In Zambia, the new first line regimen AL is far more efficacious than SP in treating uncomplicated *P. falciparum *malaria in adults. Data on safety and efficacy of AL in pregnant women are urgently needed.

## Background

In Zambia, malaria treatment and control have been undermined by the emergence of resistance to commonly-used antimalarial drugs such as chloroquine (CQ) and sulfadoxine-pyrimethamine (SP)[1]. Unacceptably high resistance to commonly used antimalarial drugs prompted the choice of artemether-lumefantrine (AL) as first line treatment for uncomplicated *Plasmodium falciparum *malaria. Zambia was the first African country to adopt an artemisinin-based combination treatment as its national policy. The safety of AL has been extensively reviewed [2]and several trials, in Africa all of them in children, have demonstrated its efficacy [3-10]. AL exists as a fixed tablet formulation and it has been registered in a large number of countries under the names of Coartem^® ^or Riamet^®^. The fixed tablet formulation helps to overcome problems of compliance associated with non-coformulated combinations.

The results of a randomized clinical trial on the efficacy of SP compared to AL in adult patients with uncomplicated malaria in Ndola, Zambia, are reported below. Patients were followed up for 45 days.

## Methods

### Study participants

The study started in March 2003, during the antimalarial drug policy transition from SP to AL, and was completed in June 2005. Patients were recruited at 4 peri-urban health centers in Ndola, Zambia, an area of mesoendemic malaria where transmission is perennial with a seasonal peak from November to April. A reliable ambulance service is available and patients requiring hospitalization can be referred to Ndola Central Hospital, about 5 km away. All individuals aged 15–50 years attending any of the 4 peri-urban clinics and presenting with fever (body temperature ≥ 37.5°C), and/or history of fever in the previous 48 hours, and without any other obvious disease were screened for malaria infection (thick and thin blood film in duplicate for parasite density and species identification) and pregnancy (if applicable). Patients with a *P. falciparum *density of 25/200 WBC (assumed to be 1,000 parasites/μl) or more were included. Exclusion criteria were the following: pregnancy, severe *falciparum *malaria [11], documented intake of SP or AL or another sulfa-drug in the two weeks prior to recruitment, other cause of fever, evidence of underlying chronic diseases (cardiac, renal, hepatic, malnutrition), history of allergy to study drug or known allergy to other sulpha drugs such as cotrimoxazol, and being non-resident in the study area. Written informed consent (in English and in Bemba) was obtained prior to recruitment from each patient. The ethical and scientific committees of the Institute of Tropical Medicine, Antwerp, Belgium, and the Tropical Disease Research Centre, Ndola approved the study.

### Enrolment, treatment and follow up

Patients were randomly allocated by blocks of 10 (according to a pre-established list) to receive either SP (Fansidar^®^, Roche: 500 mg sulfadoxine/25 mg pyrimethamine tablets), single dose of 3 tablets (2.5 tablets if < 50 kg), or AL (Coartem^®^, Novartis: 20 mg artemether/120 mg lumefantrine tablets), 4 tablets immediately followed by 4 tablets at 8, 24, 36, 48 and 60 hours, taken with a creamy snack. Treatment allocation was concealed until final recruitment. SP treatment was directly observed and patients were monitored at the health centre for at least 30 minutes after treatment. For AL, the morning doses were directly observed over the 3 days of treatment, while the evening doses were given to the patients to be taken at home, and empty sachets returned as evidence of taking the drug. Paracetamol tablets (3 doses/day for 2 days) were provided, to be taken when needed.

Clinical history, signs and symptoms, body temperature were recorded and a blood sample for parasitaemia (blood slide) and for molecular analysis (on Schleicher & Schuell filter paper) were collected at days 0, 3, 7, 14, 21, 28 and 45 or at any unscheduled visit. The blood sample on filter paper was dried at room temperature and stored at -20°C with silica gel. At day 0 (before treatment), a drop of venous blood was collected for Hb measurement (HaemoCue^®^). Hb was measured again at day 14 and 45. Patients were encouraged to attend the health facility outside scheduled visits if they felt ill. Patients treated with SP and classified as failures (clinical or parasitological) were treated with AL, while those on AL received quinine. Adverse events were documented and treated accordingly.

Patients were excluded during follow up for the following reasons: self-administration of other antimalarial drugs, emergence of any concomitant febrile illness that interfered with outcome classification, development of hypersensitivity to the study drug, withdrawal of informed consent.

### Laboratory investigations

All lab technicians were blinded to the patient's identity and all patient-related parameters. Thin blood films were fixed with methanol and thin and thick blood films were stained with 10% Giemsa. At the clinic, the number of asexual *P. falciparum *parasites per 200 white blood cells (WBC) was determined. Parasite density per μl was computed taking into account the actual WBC counts. Internal quality control was organized as recommended by the WHO [11]. Hb was assessed with Haemocue^® ^and noted with a precision of 0.1 g/dl. Blood samples collected on filter paper at enrolment and during follow up were used to genotype parasite strains; only those microscopically positive after day 14 were analysed. DNA was purified as described previously [12] and a nested PCR for the analysis of two polymorphic *P. falciparum *genetic markers MSP1 and MSP2 repeat region was carried out. A recrudescent infection was defined as one that showed match in size of at least one allele for both the MSP1 and MSP2 genes between the first and second sample.

### Assessment of outcome

Outcome of treatment was defined according to the standard WHO classification (WHO 2003): Early Treatment Failure (ETF) was defined as: i) danger signs of/or severe malaria on days 1, 2 or 3 with parasitaemia; ii) parasite density at day 2 greater than at day 0; iii) parasitaemia on day 3 with axillary temperature ≥ 37.5°C and iv) parasite density at day 3 equal or greater than 25% of that at day 0. Late Clinical Failure (LCF) was defined as danger signs of/or severe malaria and/or parasitaemia with axillary temperature ≥ 37.5°C between day 4 and day 45, without having been previously classified as ETF. Late parasitological failure (LPF) was defined as reappearance of parasitaemia between day 4 and day 45 without fever and without previously meeting any of the criteria for ETF or LCF. An adequate clinical and parasitological response (ACPR) was defined as absence of parasitaemia by day 45 without previously meeting any of the criteria for ETF, LCF and LPF. The overall rate of treatment failure (Total Treatment Failure TTF) was computed as if the patient had an ETF, LCF or a LPF. Only parasitaemia confirmed by PCR as recrudescence was considered as treatment failure. Patients were also considered treatment failures if they received rescue treatment on or before day 45. As both drugs were registered and used in Zambia, no stopping rules were defined.

All adverse events (AEs) were recorded on the Case Record Form (CRF). An AE was defined as "any unfavorable and unintended sign, symptom, or disease temporally associated with the use of the drug administered". A causality assessment of the AEs was done according to the guidelines of WHO-Uppsala Monitoring Centre (WHO-UMC).

### Statistical analysis

Data were double entered and cleaned in Epi-info (version 6.04b; Centre for Disease Control and Prevention). All analyses were performed using STATA statistical analysis software package version 8 (Stata Corp., College Station, TX, USA, 2003). Proportions were compared using the χ^2 ^or Fisher's exact test (when required); Student's t-test was used for continuous variables. Paired t-test was used for within patient comparisons. All reported p-value are two-sided. Non-parametric tests (Wilcoxon or Kruskal-Wallis) were used for non-normally distributed variables. For the intention-to-treat analysis the log-rank test and the Hazard Ratio (Cox regression) were estimated after testing for proportional hazard assumption (Schoenfeld test). Data of the patients excluded or lost to follow up were censored at the time of the last recorded visit. All possible interactions up to order two were tested.

## Results

### Enrollment

Between March 2003 and June 2005, a total of 971 patients were randomized to either SP (486) or AL (485). At enrolment the 2 groups had similar demographic and clinical characteristics (Table 1). Body temperature was significantly associated with parasite density (*P *< 0.001).

**Table 1:** Baseline characteristics by treatment (%)

	**SP** **N = 486**	**AL** **N = 485**
Mean weight (Kg) (SD)	56.4 (10.1)	56.3 (9.6)
Number of women (%)	245 (50.5)	230 (47.4)
Mean age (yr) (SD)	27.0 (8.9)	26.3 (8.3)
Mean body temperature (°C) (SD)	37.2 (1.2)	37.3 (1.3)
Mean white blood cell count (n*10^9^/l) (SD)*	5.1 (1.8)	5.2 (1.8)
Mean Hb (g/l) (SD)	13.0 (2.2)	13.2 (2.3)
Mean (geometric) parasite density(/μl) (range)*	8787 (406 – 103680)	8405 (350–158894)
Gametocytes prevalence (n)(%)	16 (3.3)	18 (3.7)
	**N = 477**	**N = 481**
Weakness (%)	344 (72.1)	365 (75.9)
Headache (%)	428 (89.7)	429 (89.2)
Muscle/joint pain (%)	316 (66.2)	318 (66.2)
Dizziness (%)	176 (36.9)	177 (36.7)
Nausea (%)	190 (39.9)	180 (37.5)
Vomiting (%)	104 (21.7)	113 (23.5)
Diarrhoea (%)	62 (13.1)	59 (12.2)
Abdominal pain (%)	172 (36.1)	163 (33.8)
Heart palpitations (%)	76 (16.0)	76 (15.7)
Backache (%)	105 (22.0)	119 (24.7)
Jaundice** (%)	51 (10.7)	104 (21.7)
Pallor** (%)	6 (1.3)	1 (0.3)
Dark urine (%)	51 (10.6)	48 (9.9)

*Data on WBC were missing in 4 patients in the SP group and in 7 in the AL group. Parasite density was calculated based on the parasites/WBC ratio.

** Jaundice and pallor in 302 patients in the SP and and 301 in the AL group.

### Trial profile

By day three, 35 (3.6%) patients were lost to follow up and 120 (12.4%) by day 45; 12 patients withdrew and 9 were excluded (4 hospitalized for reasons not linked to treatment or malaria and 5 took other antimalarial drugs). The percentage of lost to follow up were similar in both treatment arms (AL: 83/486 *vs *SP: 72/485. *P *= 0.41). The main reason for withdrawal and loss to follow up (>80%) was population movement. Patients excluded or lost during follow up were younger (mean age 24.4 yr; *P *< 0.001) but other demographic and clinical characteristics were similar.

### Analysis of efficacy

SP and AL were generally well tolerated. Almost all clinical symptoms cleared faster with AL (Table 2). At day 3, the prevalence of parasitaemia (AL:3/451,0.7%; SP:65/433,15%,) (*P *< 0.0001) and fever (AL: 4/455,0.9%;SP:15/433,3,5%) (*P *= 0.007) was significantly lower in the AL than in the SP group (Table 2). PCR genotyping identified 18 (4.6% of LTF) new infections in the SP and 19 (4.7% of LTF) in the AL group and these were considered as ACPR. ETF was significantly higher in the SP (25/465) than in the AL group (2/462) (OR:13.1; 95% CI: 3.08–55.50; *P *< 0.001). SP was also a risk factor for LCF (OR: 2.55; 95% CI: 1.34–4.84; *P *= 0.004) and LPF (OR:3.18; 95%CI: 1.25–8.09; *P *= 0.02) (Table 3). Overall, total treatment failure was more than 4-fold higher in the SP (74/391; 19.3%) than in the AL (22/404; 5.4%) group (OR: 4.15; 95% CI: 2.52–6.83; *P *< 0.001) (Table 3). Average time to new infections was similar in both treatment arms (AL:41.6 days *vs *SP:40.8 days; *P *= 0.76). Average time to recrudescence was significantly different between treatment arms (AL: 34.4 days *vs *SP: 22.7 days; *P *= 0.004).

**Table 2:** Clinical and parasitological evolution during follow up by treatment.

	**Day 3**			**Day 7**			**Day 14**			**Day 28**		
	**SP**	**AL**	**P**	**SP**	**AL**	**P**	**SP**	**AL**	**P**	**SP**	**AL**	**P**
**N**	**430**	**451**		**405**	**436**		**384**	**406**		**290**	**320**	
**Fever (%)**	**3.5**	**0.9**	**0.007**	**1.0**	**1.1**	**0.55**	**1.3**	**1.0**	**0.75**	2.7	1.2	**0.19**
**Parasitaemia**	**15.0**	**0.7**	**<0.001**	**2.0**	**0.0**	**NA**	**2,**	**0;5**	**0.009**	**5.5**	**1.2**	**0.003**
Hemoglobin (mean)*(g/L)	**-**	**-**		**-**	**-**		**124**	**129**	**0.003**	**130**	**134**	**0.02**
**Gametocyte carriage (N (%))**	**33 (7.6)**	**3 (0.7)**	**<0.001**	**88 (21.6)**	**2 (0.5)**	**<0.001**	**76 (19.8)**	**0 (0)**	**NA**	**10 (4.5)**	**1 (0,3)**	**0.004**
**Gametocyte densities (range)**	**48.6 (1–4320)**	**6.2 (1–240)**	**0.62**	**63.6 (1–4480)**	**8.9 (1–80)**	**0.51**	**52.8 (1–6960)**	**0(0)**	**NA**	**154.9 (3–840)**	**160**	**NA**
**N**	**430**	**448**		**400**	**428**		**382**	**401**		**303**	**329**	
**Weakness (%)**	**46.3**	**20.5**	**<0.001**	**15.0**	**6.1**	**<0.001**	**7.1**	**3.7**	**0.04**	5.9	3.0	0.08
**Headache (%)**	**45.6**	**22.5**	**<0.001**	**18.0**	**12.2**	**0.02**	10.7	8.5	0.28	11.5	10.3	0.62
**Muscle joint pain (%)**	**20.3**	**8.2**	**<0.001**	**5.2**	**2.6**	**0.004**	3.8	2.8	0.47	2.3	2.8	0.73
**Dizziness (%)**	**20.0**	**10.8**	**<0.001**	5.2	3.0	0.11	2.1	1.0	0.21	2.7	1.2	0.18
**Nausea (%)**	**15.8**	**2.9**	**<0.001**	1.3	0.0	NA	0.8	0.5	NA	0.7	0.6	NA
**Vomiting (%)**	**5.8**	**0.7**	**<0.001**	0.5	0.2	NA	0.5	0.5	NA	0.0	0.3	NA
**Diarrhoea (%)**	**3.8**	**7.7**	**0.013**	0,9	2.5	0.08	1.3	2.7	0.16	0.7	1.2	0.78
**Abdominal pain (%)**	**16.7**	**9.2**	**<0.001**	7.0	4.0	0.06	3.4	3.5	0.94	5.0	3.7	0.41
**Hear palpitations (%)**	**9.8**	**5.1**	**0.008**	5.5	4.2	0.38	5.8	8.0	0.61	4.6	7.3	0.88
**Back ache (%)**	**10.9**	**8.9**	0.32	7.0	7.7	0.53	5.8	8.0	0.22	4.6	7.3	0.15

In bold P value < 0,05; Other symptoms had low frequency or were not significantly different at day 3. Fisher exact (2-tailed) used where indicated. No significant differences at day 45 (data not shown). NS = not significant/NA = not applicable. * Haemoglobine day 14 and day 45 (P value: Barlett's test)

**Table 3:** PCR corrected clinical and parasitological failure at day 45 by treatment.

**Treatment outcome n/N (%)**	**SP**	**AL**	**OR (95% CI)**	**P-value**
ETF	25/465 (6.4)	2/462 (0.5)	13.1 (3.08–55.50)	<0.001
LCF	33/391 (8.4)	14/404 (3.5)	2.55 (1.34–4.84)	0.004
LPF	18/391 (4.6)	6/404 (1.5)	3.18 (1.25–8.09)	0.02
TTF (PCR corrected)	76/391 (19.3)	22/404 (5.4)	4.15 (2.52–6.83)	<0.001

Log-rank test and Cox regression gave identical results to the per protocol analysis (Figure 2) (HR: 3.77; 95 % CI, 2.34 to 6.06; *P *< 0.001). Gametocyte carriage during follow up was also significantly higher in the SP group (Table 2). By day 14 the mean Hb had decreased in both groups: SP: -6.0 g/l (paired t-test: *P *< 0.001); AL: -2.3 g/l (*P *= 0.003). At day 45 mean Hb was significantly higher in the AL (134 g/l) than in the SP (130 g/l) group (*P *= 0.02). By day 45, compared to day 0, Hb had significantly increased in the AL (+ 2.7 g/l) (*P *= 0.02) but not in the SP group (- 0.5 g/l; *P *= 0.65) (Table 2).

Serious adverse events were observed in 2 patients treated with SP (one hospitalized for severe headache received quinine and one developed hypoglycemia) and one treated with AL who developed a rash. Eleven patients (5 on AL and 6 on SP) had various symptoms possibly related to the study drugs but none serious enough to interrupt the treatment.

## Discussion

This trial was conducted in Zambia where a six-dose regimen of AL has been recently implemented as the first line treatment for uncomplicated malaria in non-pregnant adults and children over 10 kg. However, SP was still the standard therapy in March 2003 and this treatment was used as control. AL was clearly better than SP for several outcomes such as parasitological clearance, resolution of symptoms and safety. Hb followed a similar pattern, the drop observed at day 14 was more pronounced and the hematological recovery by day 45 lower in the SP arm. As expected, SP-treated patients had a significantly higher prevalence of gametocytes even if some patients treated with AL had gametocytes at day 28. These results confirm previous findings [3-7,13,14] and support the policy change with the 6-dose regime of AL recently implemented in Zambia.

Despite the high cure rate, a similar rate of new infections was observed in both treatment groups. This is a concern as routine services do not make any distinction between recrudescence and new infections. In this mesoendemic study area, the ratio new infections/recrudescence was relatively low but in places where the malaria transmission is more intense it could increase substantially. The new infections and the residual number of parasites of the 'old' infection would be exposed to sub therapeutic doses of lumefantrine, the long acting partner drug in the AL combination, and this might increase the selection of resistant strains [3]. Furthermore, low compliance might also contribute to the selection of resistant strains [15]. Indeed, a recent report from Zambia showed that even where drugs were freely available and clinic staff knew they were being observed, only 22% of patients eligible for ACTs actually received them [16]. Furthermore, even if patients receive AL, they might not comply correctly unless the importance of respecting the dosage is fully explained to them [16]. It has been shown that AL treatment, can have high cure rate irrespective of whether given under supervision with food or under conditions of routine clinic practice [17]. The recently observed increased tolerance to AL [18] underlines the importance of setting up a good surveillance system and raises questions about the useful therapeutic life (UTL) of this ACT combination. Indeed, it has already been suggested that the optimal future ACT combination might be a 3-drug ACT including 2 synergistic quinolone drugs with similar, relative long-lives protecting each other's efficacy after the fast elimination of the artemisinin derivative [19].

In Zambia, HIV-1 prevalence is estimated at 25.2 % among mothers attending the antenatal clinic [20]. HIV-1 infected adults have a higher risk of malaria infection, clinical malaria and treatment failure and this is inversely related to the absolute CD4 cells count [21-23]. Furthermore, some classes of antiretrovirals (protease inhibitors) might interfere with the physiopathology of malaria or with the metabolic pathways of antimalarial drugs [24]. In areas where both diseases are highly endemic this might have an impact on the UTL of a newly introduced ACT such as AL. The need to improve absorption of AL by co-administering it with a fatty meal and a three day, twice daily, regimen remains a concern in resource-poor settings.

## Conclusion

In conclusion, this study, despite the incompletely supervised treatment, confirmed the excellent efficacy and safety/tolerability of the six-dose AL in adults. Next to pharmacovigilance, further research is still needed to ensure its correct deployment, possibly to pregnant women.

## Authors' contributions

Modest Mulenga organized the collection of data, supervised the trial and contributed to the data interpretation and writing the paper. Jean-Pierre Van geertruyden contributed to the analysis plan, data interpretation, produced the final dataset, did the analysis and wrote the paper. Lawrence Mwananyanda organized the collection of data, supervised the trial and contributed to the data interpretation and writing the paper, Victor Chalwe supervised the trial and contributed to the data interpretation, Filip Moerman contributed to the study design and training. Roma Chilengi contributed to the study design and training. Chantal Van Overmeir and Jean-Claude Dujardin organized the PCR essays. Umberto D'Alessandro contributed to the study design, the analysis plan, data interpretation and the writing of the paper.

**Figure 1:**
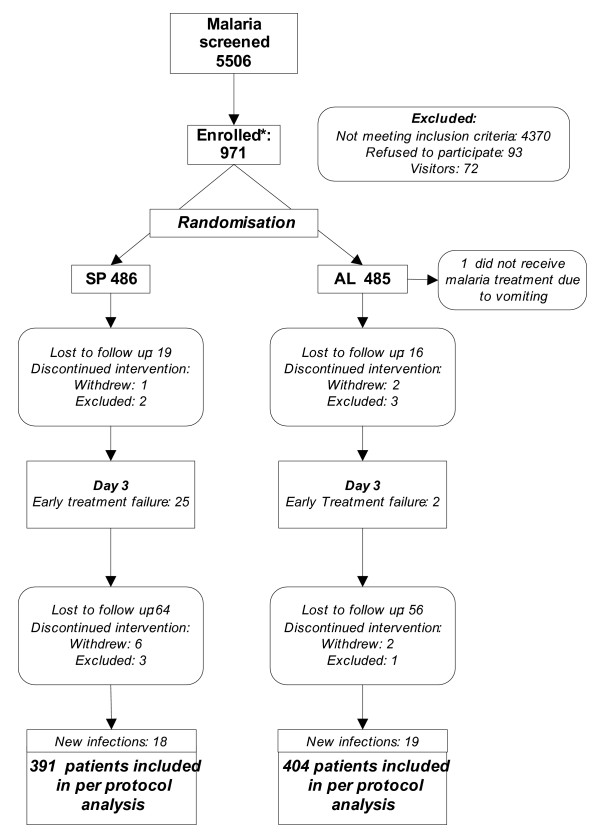
Trial profile. Legend: † 2 ETF in HIV-1 were serious adverse events. †† Patients facing an event (excluded, lost to follow up or new infection) were censored at the time of the last recorded visit and included in the survival analysis.

**Figure 2:**
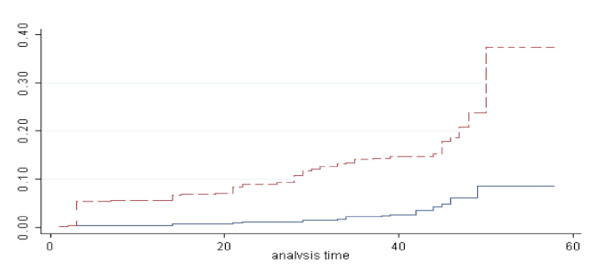
The cumulative risk of recrudescence in adults with non-complicated malaria (N = 971). All patients were censored at their last visit. A dotted line represents sulfadoxine-Pyrimethamine and a solid line Artemether-Lumefantrine (Zambia 2005).
